# A dataset of microscopic peripheral blood cell images for development of automatic recognition systems

**DOI:** 10.1016/j.dib.2020.105474

**Published:** 2020-04-08

**Authors:** Andrea Acevedo, Anna Merino, Santiago Alférez, Ángel Molina, Laura Boldú, José Rodellar

**Affiliations:** a Department of Biochemistry and Molecular Genetics. Biomedical Diagnostic Center. Clinic Hospital of Barcelona, Spain; b Department of Mathematics. Technical University of Catalonia. Barcelona East Engineering School*,* Spain; c Department of Applied Mathematics and Computer Science. Universidad del Rosario, Bogotá, Colombia

**Keywords:** Hematological diagnosis, Blood cell morphology, Blood cell images, Blood cell automatic recognition, Machine learning, Deep learning

## Abstract

This article makes available a dataset that was used for the development of an automatic recognition system of peripheral blood cell images using convolutional neural networks [1]. The dataset contains a total of 17,092 images of individual normal cells, which were acquired using the analyzer CellaVision DM96 in the Core Laboratory at the Hospital Clinic of Barcelona. The dataset is organized in the following eight groups: neutrophils, eosinophils, basophils, lymphocytes, monocytes, immature granulocytes (promyelocytes, myelocytes, and metamyelocytes), erythroblasts and platelets or thrombocytes. The size of the images is 360 × 363 pixels, in format jpg, and they were annotated by expert clinical pathologists. The images were captured from individuals without infection, hematologic or oncologic disease and free of any pharmacologic treatment at the moment of blood collection.

This high-quality labelled dataset may be used to train and test machine learning and deep learning models to recognize different types of normal peripheral blood cells. To our knowledge, this is the first publicly available set with large numbers of normal peripheral blood cells, so that it is expected to be a canonical dataset for model benchmarking.

Specifications table

**Table:** 

Subject	Hematology
Specific subject area	Computational tools for hematological diagnosis using microscopic cell images and automatic learning methods.
Type of data	Images
How data were acquired	Digital images of normal peripheral blood cells were obtained from samples collected in the Core Laboratory at the Hospital Clinic of Barcelona. In order to obtain the all blood counts, blood samples were analysed in the Advia 2120 instrument. Next, the smear was automatically prepared using the slide maker–stainer Sysmex SP1000i with May Grünwald-Giemsa staining. Then, the automatic analyser CellaVision DM96 was used to obtain individual cell images with format jpg and size 360 × 363 pixels. Images obtained were labelled and stored by the clinical pathologists.
Data format	Raw
Parameters for data collection	The dataset images were obtained from normal individuals and blood cells have been selected based on normal laboratory data.
Description of data collection	The images were collected in a 4-year period (2015 to 2019) within a daily routine. Blood cell images were annotated and saved using a random number to remove any link to the individual data, resulting in an anonymized dataset.
Data source location	Institution: Hospital Clinic of Barcelona City/Town/Region: Barcelona, Catalonia Country: Spain
Data accessibility	The dataset is stored in a Mendeley repository: Repository name: “A dataset for microscopic peripheral blood cell images for development of automatic recognition systems” Data identification number: 10.17632/snkd93bnjr.1 Direct URL to data: https://data.mendeley.com/datasets/snkd93bnjr/draft?a=d9582c71-9af0-4e59-9062-df30df05a121
Related research article	Author's name: Andrea Acevedo, Anna Merino, Santiago Alférez, Laura Puigví, José Rodellar Title: Recognition of peripheral blood cells images using convolutional neural networks. Journal: Computer Methods and Programs in Biomedicine DOI: https://doi.org/10.1016/j.cmpb.2019.105020

## Value of the data

• This dataset is useful in the area of microscopic image-based hematological diagnosis since the images have high-quality standards, have been annotated by expert clinical pathologists and cover a wide spectrum of normal peripheral blood cell types.
• The dataset can be useful to perform training and testing of machine and deep learning models for automatic classification of peripheral blood cells.
• This dataset can be used as a public canonical image set for model benchmarking and comparisons.
• This dataset might be used as a model weight initializer. This means to use the available images to pre-train learning models, which can be further trained to classify other types of abnormal cells.

## 1 Data

The normal peripheral blood dataset contains a total of 17,092 images of individual cells, which were acquired using the analyser CellaVision DM96. All images were obtained in the color space RGB. The format and size of the images is jpg and 360 × 363 pixels, respectively, and were labelled by clinical pathologists at the Hospital Clinic.

The dataset is organized in eight groups of different types of blood cells as indicated in Table 1.

**Table 1:** Types and number of cells in each group.

CELL TYPE	TOTAL OF IMAGES BY TYPE	%
neutrophils	3329	19.48
eosinophils	3117	18.24
basophils	1218	7.13
lymphocytes	1214	7.10
monocytes	1420	8.31
immature granulocytes		
(metamyelocytes, myelocytes and promyelocytes)	2895	16.94
erythroblasts	1551	9.07
platelets (thrombocytes)	2348	13.74
Total	17,092	100

Although the group of immature granulocytes includes myelocytes, metamyelocytes and promyelocytes, we have kept all in a single group for two main reasons: (1) the individual identification of specific subgroups does not have special interest for diagnosis; and (2) morphological differences among these groups are subjective even for the clinical pathologist.

**Fig. 1:**
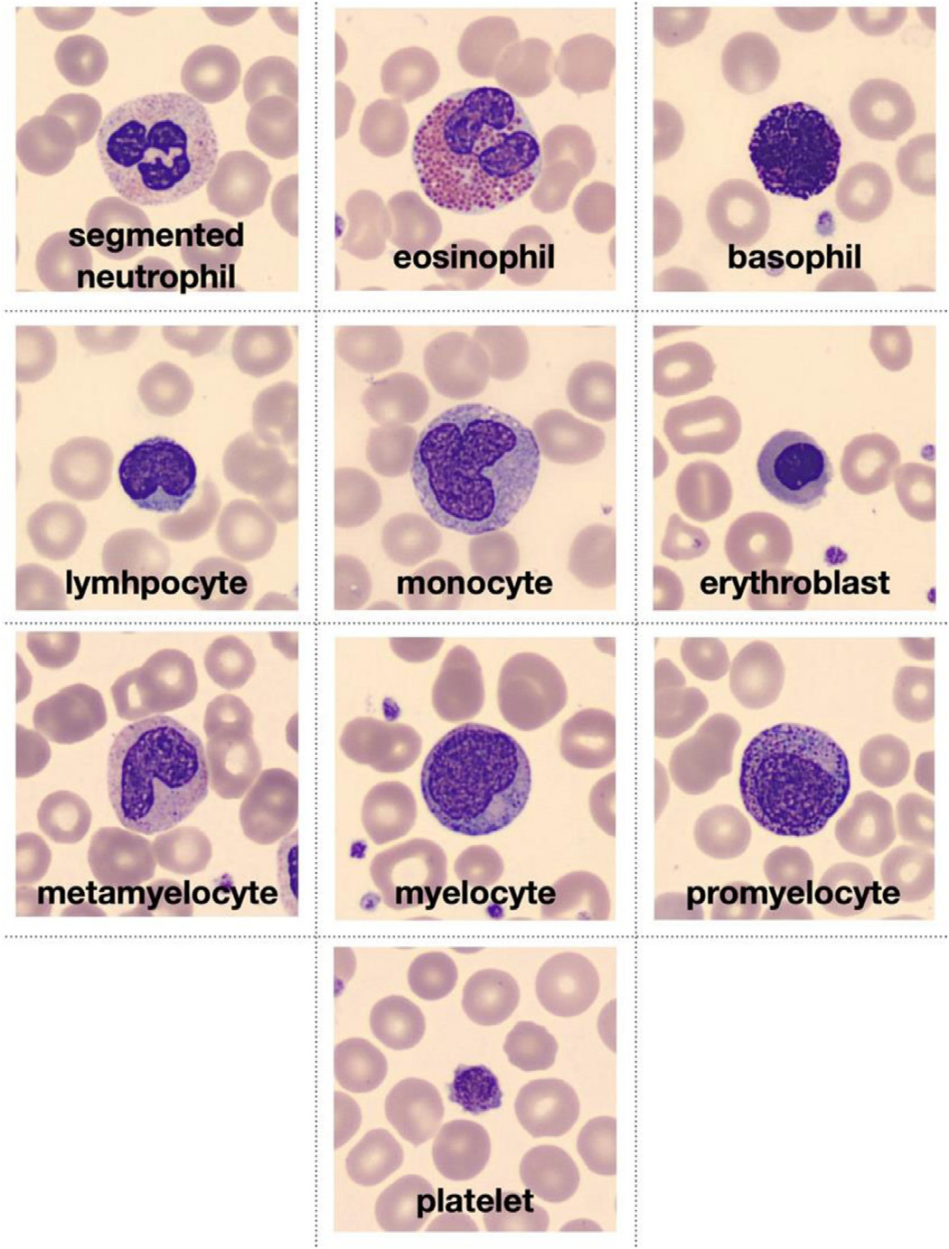
Example images of different types of normal peripheral blood cells that can be found in the dataset and organized in eight groups, including those more frequently observed in infections and regenerative anaemias.

Fig. 1 shows examples of the ten types of normal peripheral blood leukocytes that conform the dataset.

## 2 Experimental design, materials, and methods

**Fig. 2:**
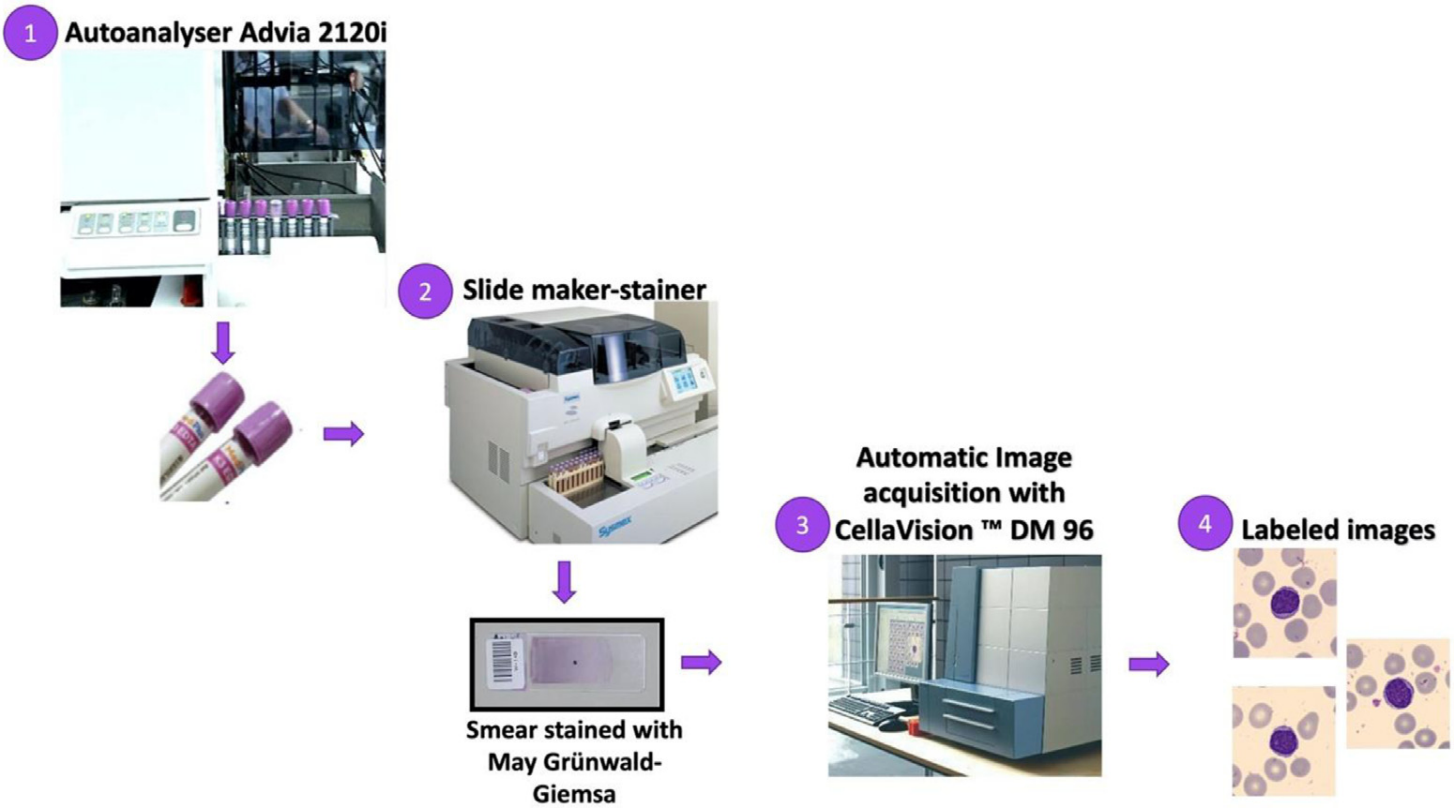
Daily work flow at the Core Laboratory performed to obtain the peripheral blood cell images.

The images were obtained during the period 2015–2019 from blood smears collected from patients without infections, hematologic or oncologic diseases and free of any pharmacologic treatment at the moment of their blood extraction. The procedure followed the daily work flow standardized in the Core Laboratory at the Hospital Clinic of Barcelona, which is illustrated in Fig. 2.

The work flow starts in the Autoanalyzer Advia 2120 instrument where blood samples are processed to obtain a general cell count. In a second step, the blood smears were automatically stained using May Grünwald-Giemsa [2] in the autostainer Sysmex SP1000i. This automated process ensures equal and stable staining regardless of the specific user. The laboratory has a standardized quality control system to supervise the procedure.

Then the resulting stained smear goes through the CellaVision DM96 where the automatic image acquisition was performed. As a result, images of individual normal blood cells, with jpg format and size 360 × 363 pixels, were obtained. Each cell image was annotated by the clinical pathologist and saved with a random identification number to remove any link and traceability to the patient data, resulting in an anonymized dataset. No filter and further pre-processing were performed to the images.

The above acquisition procedure has been extensively used by our research group in several developments related to cell image segmentation and classification of peripheral blood cells [3], [4], [5], [6], [7]. The dataset presented in this article has been used in our more recent work to develop a convolutional neural network model for the automatic classification of eight types of normal peripheral blood cells [1].

## 3 Disclaimer

This dataset is intended to be used for research and educational purposes only.

## Conflict of Interest

The authors declare that they have no known competing financial interests or personal relationships that could have appeared to influence the work reported in this paper.

## Appendix Supplementary materials

Image, application 1
